# Piperine Inhibits TGF-β Signaling Pathways and Disrupts EMT-Related Events in Human Lung Adenocarcinoma Cells

**DOI:** 10.3390/medicines7040019

**Published:** 2020-04-08

**Authors:** Leonardo Marques da Fonseca, Lucas Rodrigues Jacques da Silva, Jhenifer Santos dos Reis, Marcos André Rodrigues da Costa Santos, Victoria de Sousa Chaves, Kelli Monteiro da Costa, Julliana de Nazareth Sa-Diniz, Celio Geraldo Freire de Lima, Alexandre Morrot, Tatiany Nunes Franklim, Douglas Chaves de Alcântara-Pinto, Marco Edilson Freire de Lima, Jose Osvaldo Previato, Lucia Mendonça-Previato, Leonardo Freire-de-Lima

**Affiliations:** 1 Laboratório de Glicobiologia, Instituto de Biofisica Carlos Chagas Filho, Universidade Federal do Rio de Janeiro, Rio de Janeiro RJ 21941-902, Brazil; lfonseca@biof.ufrj.br (L.M.d.F.); lucasrjs@hotmail.com (L.R.J.d.S.); jhnffrrs8@gmail.com (J.S.d.R.); rodrigues8mr@gmail.com (M.A.R.d.C.S.); sousavictoria97@gmail.com (V.d.S.C.); kellimc85@gmail.com (K.M.d.C.); jullianawinners@gmail.com (J.d.N.S.-D.); celio@biof.ufrj.br (C.G.F.d.L.); previato@biof.ufrj.br (J.O.P.); 2 Faculdade de Medicina, Universidade Federal do Rio de Janeiro, Rio de Janeiro RJ 21941-902, Brazil; alexandre.morrot@ioc.fiocruz.br; 3 Instituto Oswaldo Cruz, FIOCRUZ, Rio de Janeiro RJ 21040-360, Brazil; 4 Instituto de Química, Universidade Federal Rural do Rio de Janeiro, Seropédica RJ 23851-970, Brazil; tnfranklim@gmail.com (T.N.F.); douglasdoti@hotmail.com (D.C.d.A.-P.); marco@ufrrj.br (M.E.F.d.L.)

**Keywords:** piperine, cancer, piperidinyl amide, epithelial–mesenchymal transition, ERK1/2, SMAD

## Abstract

**Background:** Piperine, an amide extracted from the Piper spices, exhibits strong anti-tumor properties. However, its effect on the epithelial–mesenchymal transition (EMT) process has never been investigated. Herein, we evaluate the toxic effect of piperine on lung adenocarcinoma (A549), breast adenocarcinoma (MDA-MB-231) and hepatocellular carcinoma (HepG2) cell lines, as well as its ability to inhibit EMT-related events induced by TGF-β1 treatment. **Methods:** The cell viability was investigated by MTT assay. Protein expression was evaluated by Western blot. Gene expression was monitored by real-time PCR. Zymography assay was employed to detect metalloproteinase (MMP) activity in conditioned media. Cell motility was assessed by the wound-healing and phagokinetic gold sol assays. **Results:** The results revealed that piperine was cytotoxic in concentrations over 100 µM, showing IC50 values for HepG2, MDA-MB-231 and A549 cell lines of 214, 238 and 198 µM, respectively. In order to investigate whether piperine would reverse the TGF-β1 induced-EMT, the A549 cell line was pretreated with sublethal concentrations of the natural amide followed by the addition of TGF-β1. Besides disrupting EMT-related events, piperine also inhibited both ERK 1/2 and SMAD 2 phosphorylation. **Conclusions:** These results suggest that piperine might be further used in therapeutic strategies for metastatic cancer and EMT-related disorders.

## 1. Introduction

**Figure 1:**
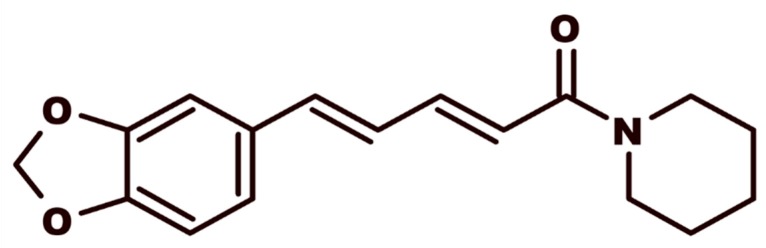
Chemical structure of piperine {1-[5-(1,3-benzodioxol-5-yl)-oxo2,4-pentadienyl]piperidine}.

The discovery of new cancer drugs is a hot topic in cancer research. Over the last twenty years, numerous studies have shown that many natural products display chemoprotective properties against different types of cancers [1,2]. In this context, piperine (a trans–trans isomer of 1-piperoyl piperidine) (Figure 1), the pungent amide present in *Piper* spices, such as the widely used black pepper (Piper nigrum L.,) is known to present a broad spectrum of biological activities, including its anti-cancer effect [3,4,5].

Regarding the multidrug resistance (MDR) phenotype, a serious obstacle for the treatment of cancer patients [6], previous studies have demonstrated that piperine inhibits the activity of the main ABC transporters related to MDR phenotype [7,8]. Since piperine has been used as a bioavailability enhancer, it also has an effect over major drug-metabolizing enzyme CYP3A4 [9,10]. Piperine mitigates liver damage prompted by renal ischemia-reperfusion [11] and ameliorates oxidative stress [12,13]. Piperine also has antipyretic [14], analgesic [15], anti-parasitic [16,17] and anti-inflammatory [15,18,19] activities. The anti-tumoral properties are particularly noteworthy because of the current interest in identifying useful natural products for cancer treatment.

Several papers have demonstrated that piperine is cytotoxic for different types of human and mouse cancer cells, including both human aggressive triple negative cell lines MDA-MB-468 and MDA-MB-231, as well as the murine cell line, 4T1 [20,21,22,23,24,25], all of which have found extensive use as in the study of molecular mechanisms related to breast cancer metastasis [26,27]. Selvediran and colleagues [28] showed that benzo(α)pyrene-induced lung carcinogenesis in mice is prevented by the oral administration of piperine. In addition, piperine-treated mice showed a decrease in lung metastasis induced by mouse mammary carcinoma and melanoma cells [25,28]. Angiogenesis was also repressed by piperine [29], which may contribute to and/or explain its anti-metastatic effect. Recent findings have demonstrated that piperine modulates cancer cell motility [22], as well as the expression and/or activity of metalloproteinases (MMPs) [30]. In addition, it has been shown that piperine is able to inhibit breast cancer stem cell renewal and Wnt signaling [31]. Hwang and colleagues [32] demonstrated that piperine strongly repressed the PMA-induced phosphorylation of the extracellular-signal-regulated kinase 1/2 (ERK1/2) in human cancer cells. More recently, it has been demonstrated that piperine treatment repressed the expression of α-smooth muscle actin (α-SMA), fibronectin (FN) and collagen in the pancreas and pancreatic stellate cells [18]. Furthermore, piperine inhibited the production of TGF-β in the pancreas and pancreatic stellate cells, as well as TGF-β-induced pSMAD 2/3 activation, suggesting that the natural amide ameliorates pancreatic fibrosis by inhibiting the TGF-β/SMAD 2/3 signaling pathway during chronic pancreatitis [18]. All those events are deeply associated with the epithelial–mesenchymal transition (EMT) process [33,34,35,36], a biological phenomenon that occurs throughout the development of fibrosis, wound-healing, tumor progression, as well as in the emergence of chemotherapy-resistant cancer cells [37,38]. EMT activation can be induced by different growth factors, such as TGF-β1, insulin-like growth factors (IGFs) and epidermal growth factors (EGF), among others [39,40]. Over the last ten years, TGF-β1 has been described as a potent EMT inducer [41]. Its role in modulating the activation of both canonical (SMAD-dependent) and noncanonical (SMAD-independent) signaling pathways is well described [42]. Since the activation of these signaling pathways is closely associated with the acquisition of a more invasive phenotype by tumor cells, the identification and/or inhibition of molecular targets associated with them is essential to fight the progression of metastatic diseases [43].

The anti-metastatic effect of piperine was first described in a murine model of lung metastasis using the B16-F10 cell line [44]. Further, Lai et al. [25] confirmed this effect in a murine breast cancer model. However, the molecular mechanism responsible for the anti-metastatic property of piperine is still unknown.

Several groups have already studied the cytotoxic effects of piperine, as well as its ability to overcome the MDR phenotype, a multifactorial phenomenon linked to EMT process [45,46,47,48,49]. Given the evidence, it is plausible to infer that piperine may be further used as a prototype molecule for the development of new derivatives with strong anti-carcinogenic effects. Over the last five years, it has been demonstrated that EMT model represents an interesting approach to the study of the anti-carcinogenic effects of natural compounds [50,51]. Although piperine is able to inhibit the events associated with cancer development and/or progression [21,22,30], its role in EMT is still unrecognized.

## 2. Materials and Methods

The antibodies against N-cadherin (N-cad; cat. number sc-59987; dilution 1:1000), Fibronectin (FN; cat. number sc-8422; dilution 1:2000), total SMAD 2 (cat. number sc-393312; dilution 1:500), p-ERK1/2 (cat. number sc-81492; dilution 1:500), total ERK1/2 (cat. number sc-514302; dilution 1:1000), MMP2 (cat. number sc-13594; dilution 1:1000), MMP-9 (cat. number sc-21733; dilution 1:1000) and Glyceraldehyde 3-phosphate dehydrogenase (GAPDH, cat. number sc-32233; dilution 1:5000) were purchased from Santa Cruz Biotechnology (Santa Cruz, CA, USA). Human TGF-β1 1 was purchased from R&D Systems (Minneapolis, MN, USA). Antibody against p-SMAD 2 (cat. number 3108; dilution 1:500) was purchased from Cell Signaling (Danvers, MA, USA). TGF-β receptor I (TGF-β 1RI; cat. number ab31013, dilution 1:1000) and TGF-β receptor II (TGF-β 1RII, cat. number ab61213, dilution 1:1000) were purchased from Abcam (Cambridge, MA, USA). Secondary antibodies HRP-conjugated anti-mouse IgG (cat. number AP308P, dilution 1:5000) or HRP-conjugated anti-rabbit IgG (cat. number AP307P, dilution 1:5000) were purchased from Millipore (Burlington, MA, USA). Protease inhibitor (cat. number P8340), Coomassie Brilliant Blue R (cat. number B0149) and DMSO (cat. number 276855) were acquired from Sigma Aldrich (St. Louis, MO, USA). The ECL chemiluminescence kit (cat. number RPN2108) was purchased from GE healthcare (Little Chalfont, Buckinghamshire, UK). PCR primers were purchased from Life Technologies (São Paulo, SP, Brazil). The alkaloid was diluted in dimethyl sulfoxide (DMSO), and 0.5%, which is not toxic to human cell line cultures [52], and this was the highest final concentration of DMSO used in the biological assays.

### 2.1. Isolation of Natural Piperine

Natural piperine used in this work was isolated from dry *P. nigrum* fruits, as previously described [51,52,53]. The amide was fully characterized through ^1^H and ^13^C nuclear magnetic resonance (NMR) spectra (Supplementary Figures S1–S3). The purity grade of isolated piperine was determined as ≥ 98% by reversed-phase high performance liquid chromatography (RP-HPLC) (Supplementary Figure S4).

The ^1^H and ^13^C nuclear magnetic resonance (NMR) spectra were recorded in a Bruker Ultrashield Plus Spectrometer (BrukerBioSpin GmbH, Rheinstetten, Germany) operating at 500 MHz for ^1^H and 125 MHz for ^13^C. ^1^H and ^13^C NMR shifts (δ) are reported in parts per million (ppm) with respect to CDCl3 (δ 7.29 ppm for 1H; and δ 77.0 ppm for 13C). Thin layer chromatography analysis was performed on silica gel pre-coated TLC Aluminum sheets, comparing with authentic samples and the spots were visualized under UV light at 254 or 356 nm). Reversed phase high performance liquid chromatography (RP-HPLC) was performed in a Shimadzu chromatograph consisting of two LC-20AT series pumps, SPD-M20A series diode array detector, and Rheodyne 7125i injector with 20 μL loop. Equipment control and data acquisition were realized using the LCSolution software (software version 1.21, Shimadzu, Kioto, Japan). Analyses were performed in a C-18 reverse phase analytical column of 150 × 4.6 mm, 5 mm of particle (Allure Restek, Bellefonte, PA, USA), maintained at 30 °C. The mobile phase used was a mixture of acetonitrile (98%, solvent B) and water (2%, solvent A). The injection volume was 20 μL and the separation was performed in isocratic mode (constant flow of 1.2 mL·min^−1^).

### 2.2. Cell Lines and Cell Culture

The human cancer cell lines used in this study were: (i) lung adenocarcinoma (A549), (ii) mammary adenocarcinoma (MDA-MB-231) and (iii) hepatocellular carcinoma (HepG2). All cell lines were obtained from the American Type Culture Collection (Manassas, VA, USA), and cultured in DMEM (Gibco, Grand Island, NY, USA) supplemented with 10 % fetal bovine serum (FBS; Life Technologies, Inc., Rockville, MD, USA) and 100 U/mL penicillin, 100 μg/mL streptomycin (Life Technologies, Inc., Rockville, MD, USA). Cells were kept at 37 °C with 5 % CO_2_ in a humidified atmosphere.

### 2.3. MTT Assay

The effects of piperine on the cell viability was evaluated through the 3-(4,5-dimethylthizol-2-yl)-2,5-diphenyltetrazolium bromide (MTT) assay as previously described [53]. Briefly, the cell lines were seeded onto a 96-well plate at a concentration of 4.0 × 10^3^ cells/well. After 18 h, the medium was renewed, and the cells were treated with increasing concentrations of piperine (20, 40, 80, 160, 320 µM) for the next 72 h. At the end of the exposure period, the cells were incubated with 20 μL of MTT solution (5 mg/mL) (Sigma Chemical Co., St. Louis, MO, USA) for 4 h at 37 °C. After the medium was removed, 100 μL of DMSO was added to each well, and the absorbance was measured with a plate reader (Model AD340, Beckman Coulter, Brea, CA, USA) at a wavelength of 570 nm. The cell viability index was calculated using the following formula: experimental optical density value/control OD value. Each experiment was repeated three times.

### 2.4. Cell Treatment for EMT Analysis

A549 cells (4 × 10^5^ cells per well) were plated in 6-well plates. After 18 h, the medium was changed, and the cell monolayers were pre-treated or not with increasing concentrations of piperine (20, 40, 80 μM) for 24 h. After pretreatment, the cells were stimulated or not with 1 ng/mL TGF-β1, and the cell cultures incubated for the next 48 h at 37 °C with 5 % CO_2_ in a humidified atmosphere.

### 2.5. Immunoblotting Assay

A549 cells were plated and treated or not with piperine and TGF-β1, as described in Section 2.4 of the Materials and Methods. After treatment, the cells were scrapped and lysed in RIPA buffer (50 mM Tris-HCl pH 7.4; 0.5% NP-40; 250 mM NaCl; 5 mM EDTA and 50 mM NaF) containing freshly added protease inhibitor solution [54]. Protein content was determined by using a microBCA protein assay reagent kit (Pierce), with BSA as standard. Aliquots (30 μg of protein per lane) were subjected to SDS/PAGE and transferred to nitrocellulose membranes. Blocking was performed overnight with Tris-buffered saline with 0.1% (v/v) Tween 20 (TBS-T) containing 5% (w/v) nonfat dry milk. The membranes were incubated with primary antibodies for 2 h at room temperature and, after several washes with TBS-T, incubated for one more hour with the appropriate secondary antibody and then developed using an ECL chemiluminescence kit (GE Healthcare, USA). ImageJ software was used for densitometry analysis of immunoblots, and all measurements were normalized against GAPDH loading controls [55].

### 2.6. Cell Morphology and Circularity Analysis

A549 cells were plated and treated or not with piperine and TGF-β1 as described before. After treatment, photomicrography was taken by phase-contrast microscopy (Nikon) at 80 × magnification. Circularity ratio (C) was calculated as **C = P/(4πA)^0.5^**, where P and A are, respectively, the perimeter and area of the cell [56].

### 2.7. Cell Motility Assay

Cell motility was assessed by the wound-healing and phagokinetic gold sol assays as previously described [57]. For phagokinetic gold sol assay, cells were plated and treated or not with piperine and TGF-β1 as mentioned. After treatment, cells were detached, and 5.0 × 10^2^ cells were plated onto gold sol-coated well and incubated for 18 h. Photographs were taken by phase-contrast microscopy (Nikon) at 80 × magnification; the track area of 200 cells was measured by using the Scion Image program, and expressed as squared pixels. For the wound-healing assay, cells were treated as above and scratches were made on the cell monolayers with plastic pipette tips by moving them perpendicularly to the lines marked at the bottom of wells. The cells were then rinsed and incubated in culture medium for 18 h. Pictures were taken at 0 h and 18 h. The procedure was based on previous studies [57,58].

### 2.8. Zymography

For gelatinase activity, cell culture supernatants were used as previously described [55]. In brief, samples were subjected to SDS/PAGE, using 1.5 μg·mL^−1^ gelatin type A from porcine skin (Sigma). The gels were renatured, developed in 50 mM Tris, 2.5 mM CaCl, pH 7.5, overnight at 37 °C, stained with Coomassie Brilliant Blue R, and then destained until the bands became clear.

### 2.9. Determination of mRNA Levels by Real-Time Quantitative PCR (qRT-PCR)

The number of transcript copies was monitored by qRT-PCR analysis as previously described [56]. In brief, A549 cells were plated and treated or not with piperine and TGF-β1 as described before, and the total RNA of cells was extracted and purified using Qiagen RNeasy Mini Kit (Qiagen, Germantown, MD, USA). The cDNA was prepared from 2 µg of total RNA using a RevertAid First Strand cDNA Synthesis Kit (Thermo Fisher, Bartlesville, OK, USA) with oligo-dT primer, according to manufacturer’s instructions. qRT-PCR was performed using SYBRGreen QRT-PCR Kit plus ROX (LGC Biotecnologia, São Paulo, SP, Brazil) according to the manufacturer’s protocols. The following primer pairs were used: E-cadherin (E-cad): (sense, 5′-CGGGAATGCAGTTGAGGATC -3′; antisense, 5′-AGGATGGTGTAAGCGATGG-3′), N-cad: (sense, 5′-CTCCTATGAGTGGAACAGGAACG -3′; antisense, 5′-TTGGATCAATGTCATAATCAAGTGCTGTA-3′), FN: (sense, 5′-TTATGACGACGGGAAGAC -3′; antisense, 5′-GCTGGATGGAAAGATTAC -3′) and GAPDH: (sense, 5′-TGACTTCAACAGCGACACCCA-3′; antisense, 5′-GCCAAATTCGTTGTCATAC-3′). Amplification was carried out as previously described in Alisson-Silva et al. [56].

### 2.10. Statistical Analysis

Statistical analyses were performed using the software GraphPad Prism (Software version 7, San Diego, CA, USA). Each experiment was repeated at least three times. Data were expressed as means ± SD and were analyzed using one-way ANOVA with a Bonferroni posttest for a comparison of the differences. Values of *p* ≤ 0.05 were accepted as statistically significant.

## 3. Results 

### 3.1. Piperine Inhibits Proliferation of Human Cancer Cell Lines

**Figure 2:**
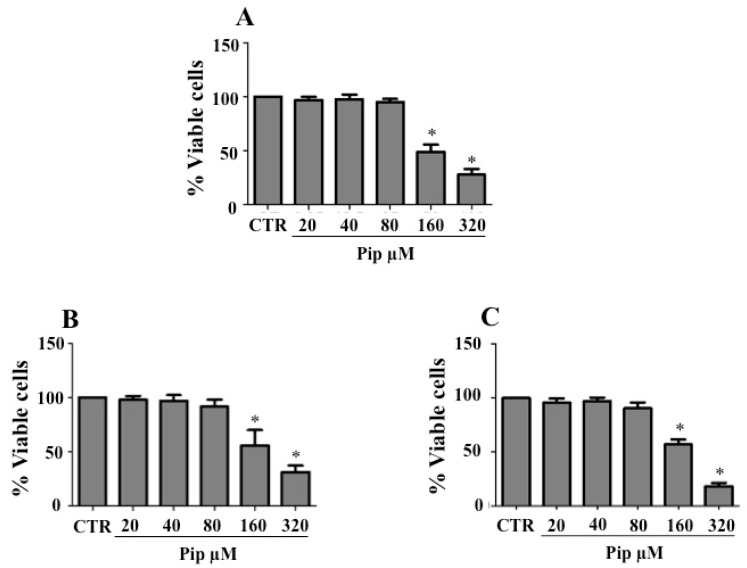
Inhibition of growth by piperine in lung adenocarcinoma (A549), breast adenocarcinoma (MDA-MB-231) and hepatocellular carcinoma (HepG2) cells. (**A**) HepG2, (**B**) MDA-MB-231 and (**C**) A549 cells were treated or not (CTR) with 20, 40, 80, 160 and 320 μM piperine for 72 h, and the cell viability was monitored by MTT assay at 570 nm using a Beckman Coulter AD 340 plate-reading spectrophotometer. The following formula has been used to calculate the percentage of viable cells: (viable cells) % = (OD of drug-treated sample/OD of untreated sample) × 100. Data are representative of three independent experiments ± SD. * *p* ≤ 0.05 vs. untreated cells (CTR).

Before assessing the effect of piperine on cancer cells undergoing EMT, a cell viability assay was performed to determine the IC_50_ and select nonlethal concentrations of the natural amide for further analysis. For this purpose, cells were treated with increasing concentrations (20–320 μM) of piperine for 72 h. The cell viability monitored by the MTT assay, showed that 160 and 320 μM of piperine were toxic for all cell lines utilized. The IC_50_ values for HepG2, MDA-MB-231 and A549 cells were 214 µM (Figure 2A), 238 µM (Figure 2B) and 198 μM (Figure 2C), respectively. Because there were no significant differences in cell viability when the concentrations of the amide were below 80 μM (Figure 2A–C), three concentrations of piperine ranging from 20 to 80 μM were selected for the following experiments.

### 3.2. Piperine Supresses Morphological and Phenotypical Changes Induced by TGF-β1

**Figure 3:**
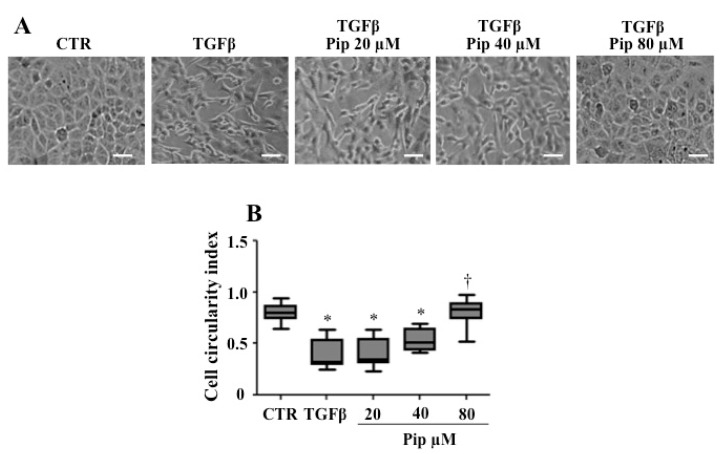
Pretreatment with piperine modulates TGF-β1-induced morphological changes in A549 cells. (**A**) A549 cells were pretreated with non-cytotoxic doses of piperine (20, 40 and 80 μM) for 24 h followed by treatment with 1 ng/mL TGF-β1 for 48 h, then followed by the observation under phase-contrast microscopy, scale bar = 100 μM. (**B**) After TGF-β1 treatment, cells acquired spindle-shaped morphology. Pretreatment with piperine 40 and 80 μM attenuated or suppressed such event, respectively. Data are representative of 500 cells from three independent experiments ± SD. * *p* ≤ 0.05 vs. untreated cells (CTR); † *p* ≤ 0.05 vs. TGF-β.

To analyze the anti-EMT effect of piperine, we used the A549 cell line, since it has been approved in vitro as an interesting model for monitoring EMT-related events [59]. As expected, when 1 ng/mL TGF-β1 was added to cell culture, cells lost cell–cell adhesion and changed from a compact epithelial morphology to a spindle-shaped cell morphology (Figure 3A). After TGF-β1 treatment, the cell circularity ratios were significantly reduced when compared with control epithelial cells, which morphologically resemble a circle, presenting circularity ratios approaching one (Figure 3B). However, when cells were incubated with sublethal concentrations of the alkaloid, especially 40 and 80 μM, prior to TGF-β1 treatment, such changes were significantly suppressed (Figure 3A). In this condition, the cell circularity was similar to control cells (Figure 3B).

**Figure 4:**
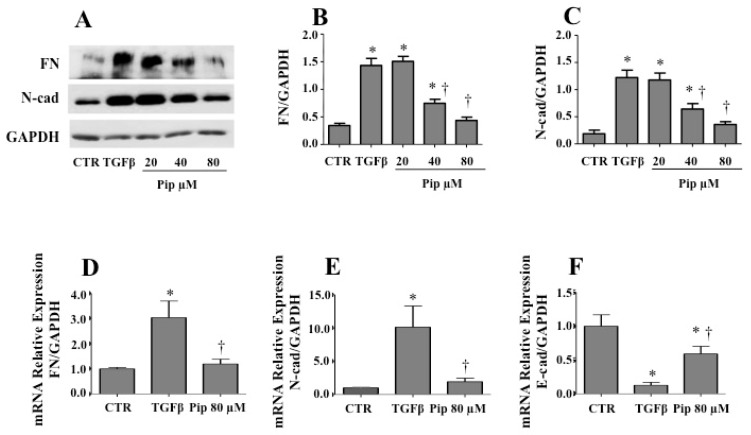
Pretreatment with piperine attenuates TGF-β1-induced mesenchymal-like phenotype in A549 cells. A549 cells were pretreated with non-cytotoxic doses of piperine (20, 40 and 80 μM) for 24 h, followed by treatment with 1 ng/mL TGF-β1 for 48 h. After treatment, cells were used for Western blot and qPCR analysis. (**A**) Western blot analysis of the expression of mesenchymal markers fibronectin (**B**) and N-cad (**C**). GAPDH was used as loading control. Densitometry was performed using the image-processing program ImageJ. The relative copy number of transcripts for fibronectin (**D**), N-cad (**E**) and E-cad (**F**) was determined by qRT-PCR to evaluate changes in gene expression. The results are representative of three independent experiments. The results are shown as mean ± SD. * *p* ≤ 0.05 vs. untreated cells (CTR); † *p* ≤ 0.05 vs. TGF-β.

The ability of piperine in modulating the cell phenotype induced by TGF-β1 was first evaluated by Western blot analysis (Figure 4). In response to TGF-β1, the expression of the mesenchymal markers FN and N-cad was significantly increased. However, when cells where pretreated with 40 and 80 μM of piperine, such events where abrogated (Figure 4A–C). qPCR results corroborated Western blot data, since the ability of TGF-β1 to up-regulate the mRNA levels for FN (Figure 4D) and N-cad (Figure 4E) were significantly repressed by piperine 80 µM (Figure 4D,E). As expected, mRNA levels for epithelial marker E-cad were considerably reduced by TGF-β1. However, such an event was attenuated by pretreatment with piperine 80 µM (Figure 4F).

### 3.3. Piperine Inhibits A549 Cell Migration and MMP-2 Secretion Induced by TGF-β1

**Figure 5:**
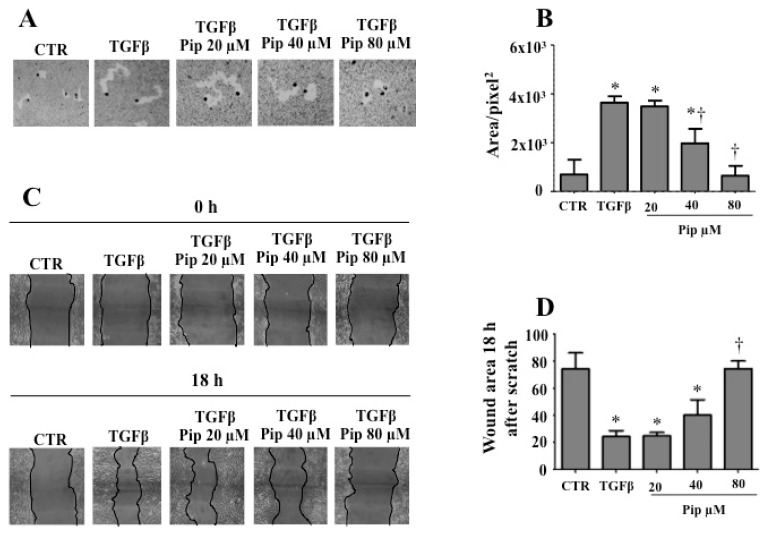
Pretreatment with piperine inhibits the cell motility of A549 cells treated with TGF-β1. To analyze the cell motility, A549 cells were pretreated with non-cytotoxic doses of piperine (20, 40 and 80 μM) for 24 h, followed by treatment with 1 ng/mL TGF-β1 for 48 h. After treatments, the cell motility was evaluated based on phagokinetic motility (**A**,**B**) and wound assay (**C**,**D**). For phagokinetic motility, track area of 200 cells was monitored with the aid of the program Scion Image and expressed as squared pixels. For wound assay, photos were taken before and after incubation. The results are representative of four independent experiments. Quantitative analyses are shown as mean ± SD. * *p* ≤ 0.05 vs. untreated cells (CTR), † *p* ≤ 0.05 vs. TGF-β.

Since the acquisition of a spindle-shaped morphology and reduced intercellular adhesion is a fundamental requirement for cell motility, we next examined the effect of piperine on the TGF-β1-induced migration of A549 cells. As expected, the phagokinetic gold sol (Figure 5A,B) and wound-healing (Figure 5C,D) assays revealed that TGF-β1 enhanced the cell motility when compared to the control cells, whereas pretreatment with 40 and 80 μM piperine inhibited the TGF-β1-induced cell migration.

**Figure 6:**
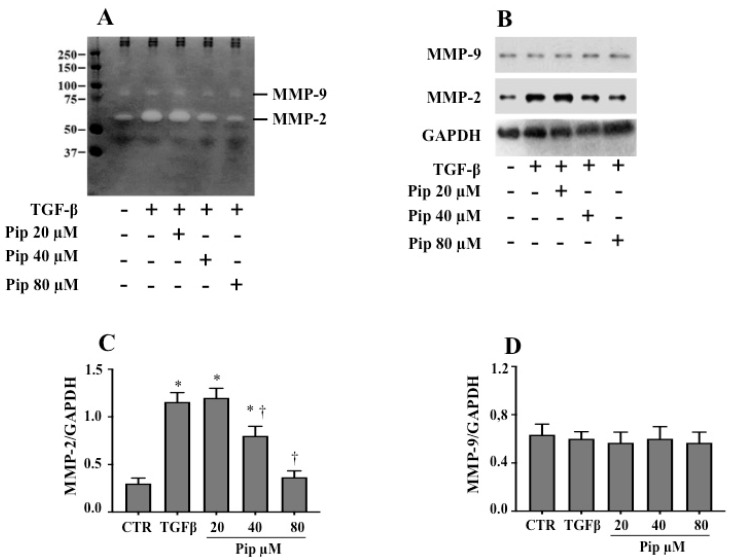
Pretreatment with piperine inhibits MMP-2 activity and expression in A549 cells treated with TGF-β1. A549 cells were pretreated with non-cytotoxic doses of piperine (20, 40 and 80 μM) for 24 h, followed by treatment with 1 ng/mL TGF-β1 for 48 h. (**A**) After treatments, gelatin zymography was employed to detect MMP activity. (**B**) Western blot analysis to investigate the expression of MMP-2 (**C**) and MMP-9 (**D**) was performed by using the cell culture medium. The maximum volume of supernatant used to analyze the activity and expression of MMPs was 30 µL. The volume of each experimental point was adjusted based on the amount of protein in the cell lysates. The results are representative of three independent experiments. Quantitative Western blot analyses are shown as mean ± SD. * *p* ≤ 0.05 vs. untreated cells (CTR), † *p* ≤ 0.05 vs. TGF-β.

Furthermore, we used gelatin zymography (Figure 6A) and Western blot (Figure 6B–D) analysis to examine the inhibitory effect of the natural amide on MMP-2 and MMP-9 activities. When compared to the control, TGF-β1-treated cells presented an elevated activity (Figure 6A) and expression (Figure 6B,C) for MMP-2. Interestingly, treatment with 40 and 80 µM piperine, prior to TGF-β1 addition, was able to suppress such phenomena (Figure 6A–C). The inhibitory effect seems to be specific for MMP-2, since no concentration of the alkaloid was able to compromise the basal MMP-9 activity and expression (Figure 6A,B,D). These results suggest that piperine prevents the acquisition of an invasive phenotype, as well as the migration of A549 cells at non-toxic concentrations.

### 3.4. Piperine Inhibits TGF-β1 -Induced Activation of ERK and SMAD Signaling Pathways

**Figure 7:**
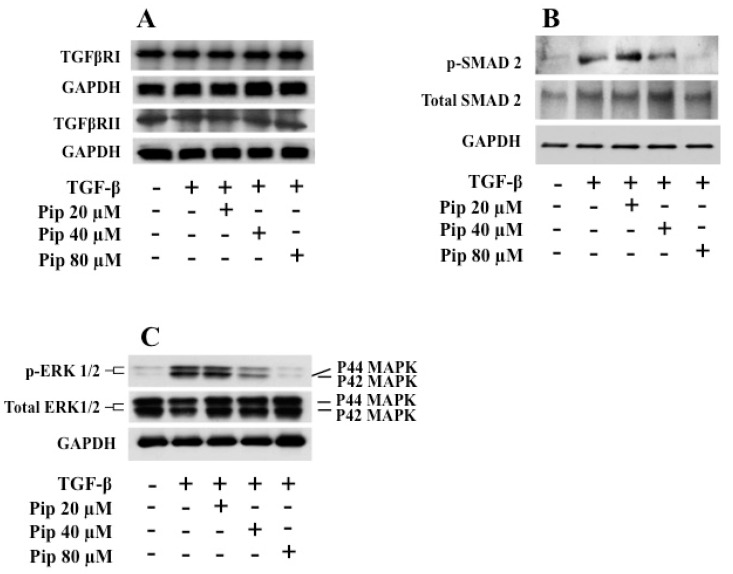
Effect of piperine on TGF-β receptors (TGFβRI and TGFβRII) and ERK 1/2 and SMAD2 activation in A549 cells treated with TGF-β1. A549 cells were plated in serum free medium and pretreated with 20, 40 and 80 μM piperine for 24 h. After treatment, cells were or not stimulated with 1 ng/mL recombinant human TGFβ1. The cells were then collected, washed with PBS and lysed in RIPA buffer. Western blot analysis was employed to evaluate (**A**) the expression of TGFβRI and TGFβRII receptors, (**B**) phospho-SMAD2 and (**C**) phospho-p44/42 MAPK (ERK1/2).

Usually, the effect of TGF-β1 on SMAD proteins does not vary in most cell types and is named the canonical TGF-β pathway. In addition to activating SMADS, TGF-β1 also modulates the activation of numerous cell-signaling pathways [60,61]. Unlike the canonical pathway, the modulation of other signaling pathways by TGF-β1 is usually dependent on the context and cell type and is known as the noncanonical TGF-β signaling pathway [62]. It has been well documented that over the last ten years, the canonical TGF-β1 signaling pathway governed the field of TGF-β research. However, more recently, increasing attention has been paid to the noncanonical TGF-β signaling, particularly in the context of EMT-related events [63,64]. In order to evaluate the signaling pathways activated during EMT induction by TGF-β1, and the effect of piperine pretreatment on them, we performed Western blot assays for TGF-β receptors, p-SMAD2 and p-ERK 1/2. Pretreatment with piperine did not reduce the expression of either type I (TGFβRI) or type II (TGFβRII) TGF-β receptors (Figure 7A). Since receptor expression was unchanged, we decided to test the downstream signaling pathways. As expected, TGF-β1 was able to increase phosphorylation of both SMAD2 and ERK 1/2 when compared to the control condition (Figure 7B,C). However, pretreatment with 40 an 80 µM piperine was able to downregulate the phosphorylation of both SMAD-2 (Figure 7B) and ERK1/2 (Figure 7C), which may explain its anti-EMT effect.

## 4. Discussion

Metastatic disease is the cause of over 90% of cancer-induced mortalities, which might be explained by the lack of effective treatments. For this reason, the concept that plant-derived natural products represent a key source of bioactive compounds remains a major challenge for both clinicians and scientists [65]. Metastasis can be divided into several stages. Among these, there is the improvement of migratory propriety, which can be attained through EMT [66,67]. TGF-β1-induced EMT in A549 cells is a widely used model to study pulmonary fibrosis and lung cancer [59]. Our results corroborate previous studies, which have shown that TGF-β1 induces A549 cells to lose their epithelial characteristics and to acquire a spindle-like appearance [68,69,70].

The anti-tumoral effect of piperine was described over fifteen years ago [44]. Since then, several studies have shown that the natural amide present potential as an anti-cancer agent, as it inhibits several aspects related to cancer progression and metastasis [71,72,73]. The EMT process is characterized by morphological, phenotypic and biochemical alterations, which together influence the behavior of tumor cells of epithelial origin [74]. In the literature, many studies have shown that piperine modulates crucial events related to EMT process, such as cell motility [22], MMP expression and activity [30], expression of mesenchymal proteins [18], as well as the activation of MAPK and SMAD signaling pathways [18,75]. However, so far, no study has demonstrated the ability of piperine to collectively compromise all of these events in cancer cells undergoing EMT.

Herein, we confirm that piperine presents potential as an anti-cancer agent, since it was able to significantly reduce the viability of cancer cells. We also observed that the administration of sublethal concentrations of piperine disrupted classical events related to TGF-β1 induced-EMT processes, such as morphology changes, phenotypic alterations, increased cell motility and expression of metalloproteinases [66,76]. In order to monitor the effect of piperine on cell motility, we initially used the wound assay, a widely used technique [55,77,78,79]. The results showed that piperine abrogated the increased cell motility induced by TGF-β1. However, since we did not use substances to block cell proliferation in this assay, the cell growth would mask the motility event. For this reason, we used another known technique, the phagokinetic gold sol assay, which has the advantage of measuring the motility of individual cells [55,80]. The results also demonstrated that piperine compromised the gain of migratory property by cancer cells.

Recently, Choi et al. [18] demonstrated that in a murine study of chronic pancreatitis, piperine attenuated the production of TGF-β in the pancreas and improved the severity of fibrosis through inhibition of TGF-β/SMAD signaling pathways. The authors also demonstrated that in TGF- β-treated pancreatic stellate cells, piperine reduced the expression of the mesenchyml markers αSMA, FN and collagen. Likewise, we also observed that the expression of mesenchymal markers (FN and N-cad) induced by TGF-β1 was abrogated when A549 cells were pretreated with piperine.

In addition, we demonstrated that piperine inhibits both canonical and noncanonical TGF-β signaling pathways in A549 cells undergoing EMT. Previous studies revealed that piperine obstructs NF-kB signal transduction cascade [81,82] and inhibits p38 [75,83], JNK [84] and ERK 1/2 [32,85] signaling pathways. Since both ERK and SMAD pathways might be activated by TGF-β1, and modulate the EMT process [35,86,87,88], their inhibition may explain the anti-EMT effect of piperine. In 2011, Li and colleagues [7] described that piperine is able to re-sensitize multidrug resistant cancer cells. Recent studies support a close connection between EMT activation, the expression of ATP binding cassette (ABC) proteins and drug resistance [88,89,90,91]. Further studies need to be performed, but it is plausible to speculate that, somehow, the inhibition of the activity and/or expression of ABC transporters might enhance the anti-EMT effect of the natural amide.

## 5. Conclusions

Our study provides the first piece of evidence that piperine can attenuate EMT-related events induced by TGF-β1 in the human alveolar adenocarcinoma A549 cells associated with inhibition of both canonical and noncanonical TGF-β1 signaling pathways. More importantly, piperine also prevents the cell from acquiring greater migratory capacity, N-cad and FN expression and MMP-2 secretion. Given the gathered data, it is reasonable to speculate that piperine may show therapeutic potential against EMT-related disorders. The continued advancement of this line of research may open the door to an entirely new class of anti-cancer drugs.

## Supplementary Materials

The following are available online at https://www.mdpi.com/2305-6320/7/4/19/s1, Figure S1: NMR ^1^H spectrum (500 MHz, CDCl_3_) of natural piperine, Figure S2: NMR ^13^C spectrum (125 MHz, CDCl_3_) of natural piperine, Figure S3: NMR ^1^H and ^13^C data for natural piperine, Figure S4: HPLC-RP for piperine (Retention time: 5.6 min.; Purity grade ≥ 98%).
