# Supplementary material for: Piperine Inhibits TGF-β Signaling Pathways and Disrupts EMT-Related Events in Human Lung Adenocarcinoma Cells
Source: Medicines (Basel). 2020 Apr 8;7(4):19. doi: 10.3390/medicines7040019 (PMC7235759; doi:10.3390/medicines7040019)
Supplement: Supplementary file 1 [file medicines-07-00019-s001.pdf]

Supplementary Materials: Piperine Inhibits TGF-β Signaling Pathways and Disrupts EMT-related Events in Human Lung Adenocarcinoma Cells

Leonardo Marques da Fonseca, Lucas Rodrigues Jacques da Silva, Jhenifer Santos dos Reis, Marcos André Rodrigues da Costa Santos, Victoria de Sousa Chaves, Kelli Monteiro da Costa, Julliana de Nazareth Sa-Diniz, Celio Geraldo Freire-de-Lima, Alexandre Morrot, Tatiany Nunes Franklim, Douglas Chaves de Alcântara-Pinto, Marco Edilson Freire de Lima, Jose Osvaldo Previato, Lucia Mendonça-Previato and Leonardo Freire-de-Lima

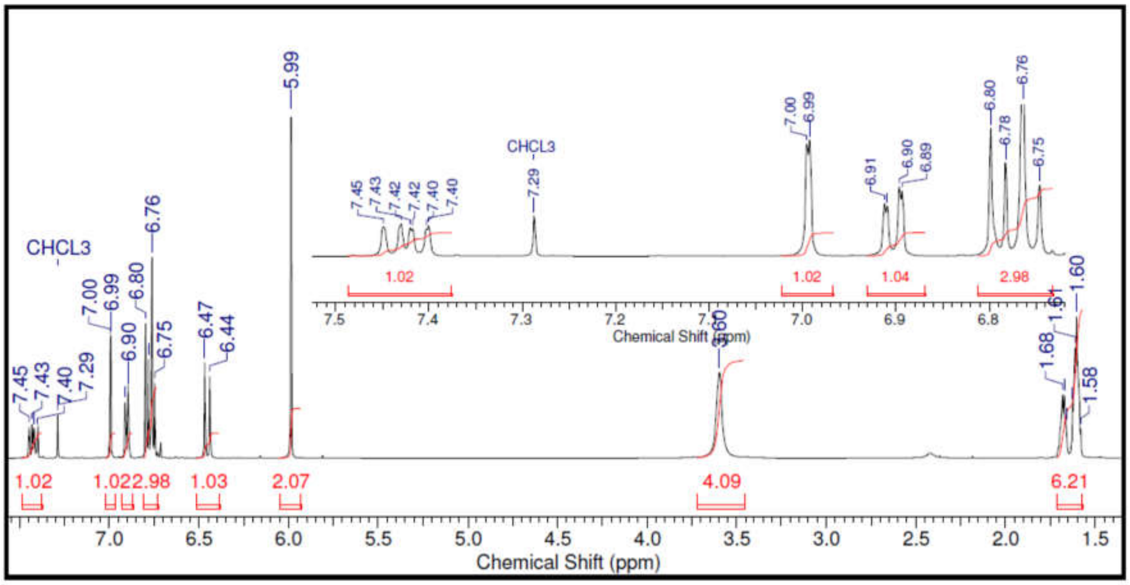

Figure S1. NMR ^1H spectrum (500 MHz, $CDCl_3$) of natural piperine.

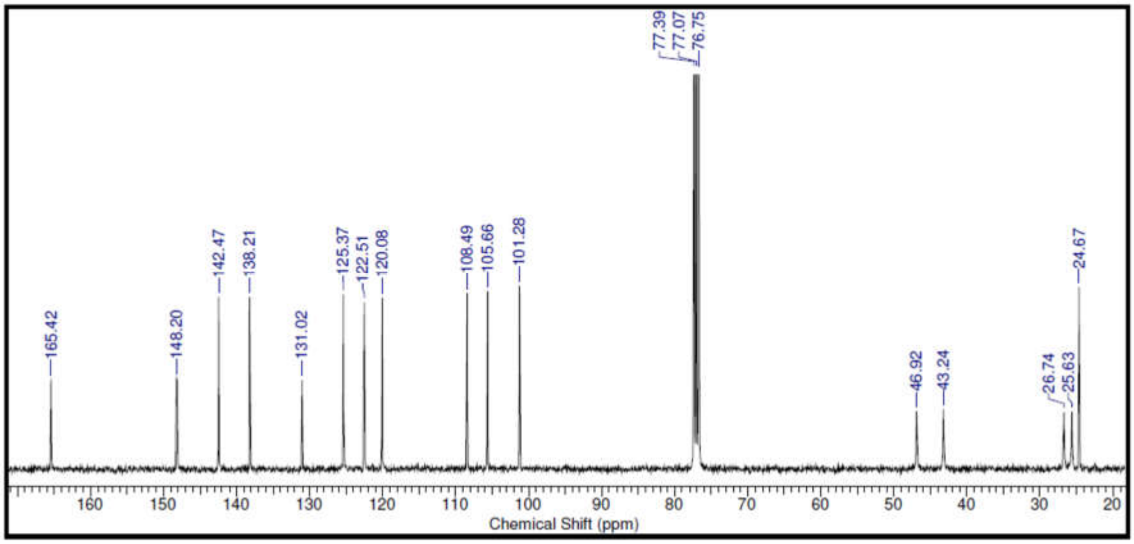

Figure 2. NMR ^{13}C spectrum (125 MHz, $CDCl_3$) of natural piperine.

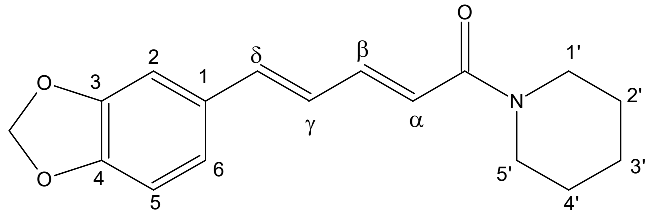

Piperine structure*[1]

Chemical shifts of ^1H NMR and ^{13}C NMR for natural piperine*[2]

Position	δ ^{1}H (ppm); *J* (Hz)*[3]	δ^{13}C (ppm)*[3]
1′	*3.60 (m, 2H)*	43.24 (CH_2)
2′	*1.58 -1.68 (m, 2H)*	24.67 (CH_2)
3′	*1.58 – 1.68 (m, 2H)*	25.63 (CH_2)
4′	*1.58 – 1.68 (m, 2H)*	26.74 (CH_2)
5′	*3.60 (m, 2H)*	46.92 (CH_2)
C=O	---------------	165.42 (C)
α	6.45 (d; 14.5 Hz; 1H)	120.08 (CH)
β	7.43 (dd; 14.5 and 9.7 Hz; 1H)	142.47 (CH)
γ	6.75 – 6.80 (m, 1H)	125.37 (CH)
δ	6.75 – 6.80 (m, 1H)	138.21 (CH)
1	---------------	131.02 (C)
2	*6.99 (d; 1.5 Hz; 1H)*	105.66 (CH)
3 e 4	---------------	148.20 (C)
5	6,75 - 6,80 (m, 1H)	108.49 (CH)
6	6.90 (dd; 8.0 and 1.5 Hz; 1H)	122.51 (CH)
OCH_2O	5.99 (s, 2H)	101.28 (CH_2)

*[1]The numbering used was oly for assignment of NMR data and does not follow any nomenclature rule. *[2]The ^1H and ^{13}C NMR spectra were obtained in $CDCl_3$ at 500 and 125 MHz, respectively. *[3]All the NMR data obtained are in accordance to that previously described in the literature for piperine.

Figure S3. NMR ^1H and ^{13}C data for natural piperine.

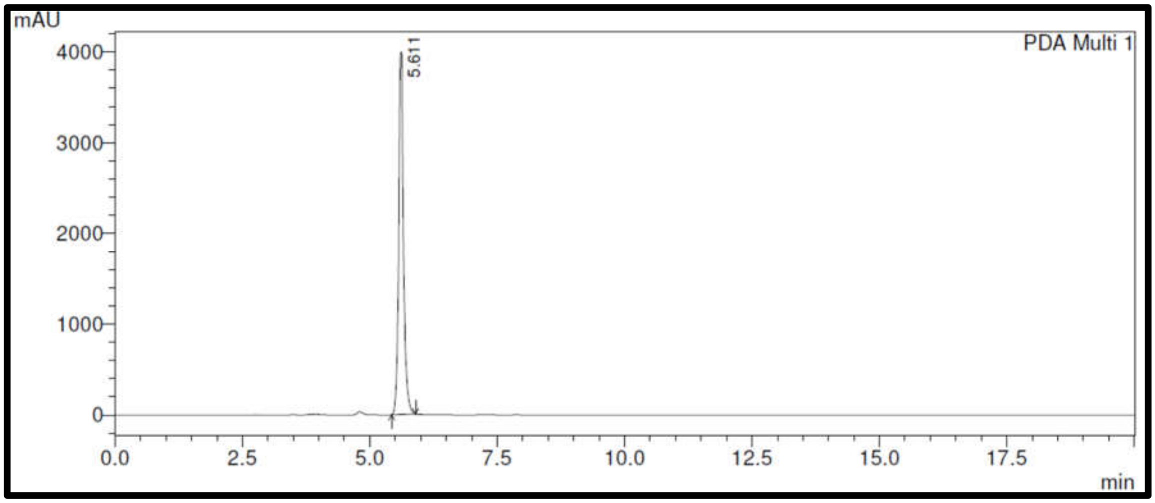

Figure S4. HPLC-RP for piperine (Retention time: 5.6 min.; Purity grade ≥ 98%).
